# Evolution of oesophageal adenocarcinoma from metaplastic columnar epithelium without goblet cells in Barrett's oesophagus

**DOI:** 10.1136/gutjnl-2015-310748

**Published:** 2015-12-23

**Authors:** Danielle L Lavery, Pierre Martinez, Laura J Gay, Biancastella Cereser, Marco R Novelli, Manuel Rodriguez-Justo, Sybren L Meijer, Trevor A Graham, Stuart A C McDonald, Nicholas A Wright, Marnix Jansen

**Affiliations:** 1 Barts Cancer Institute, Queen Mary University of London, London, UK; 2 Department of Pathology, University College London, London, UK; 3 Department of Pathology, Academic Medical Centre, Amsterdam, The Netherlands

**Keywords:** BARRETT'S CARCINOMA, BARRETT'S METAPLASIA, BARRETT'S OESOPHAGUS

## Abstract

**Objective:**

Barrett's oesophagus commonly presents as a patchwork of columnar metaplasia with and without goblet cells in the distal oesophagus. The presence of metaplastic columnar epithelium with goblet cells on oesophageal biopsy is a marker of cancer progression risk, but it is unclear whether clonal expansion and progression in Barrett's oesophagus is exclusive to columnar epithelium with goblet cells.

**Design:**

We developed a novel method to trace the clonal ancestry of an oesophageal adenocarcinoma across an entire Barrett's segment. Clonal expansions in Barrett's mucosa were identified using cytochrome c oxidase enzyme histochemistry. Somatic mutations were identified through mitochondrial DNA sequencing and single gland whole exome sequencing.

**Results:**

By tracing the clonal origin of an oesophageal adenocarcinoma across an entire Barrett's segment through a combination of histopathological spatial mapping and clonal ordering, we find that this cancer developed from a premalignant clonal expansion in non-dysplastic (‘cardia-type’) columnar metaplasia without goblet cells.

**Conclusion:**

Our data demonstrate the premalignant potential of metaplastic columnar epithelium without goblet cells in the context of Barrett's oesophagus.

**Significance of this study**

**What is already known on this subject?**

- The cancer progression risk in patients with columnar metaplasia of the oesophagus remains in doubt.
- Although the presence of goblet cells on oesophageal biopsy is an accepted progression risk marker, it is unclear whether clonal progression is exclusive to intestinalised epithelium.
- Lineage tracing through detection of shared somatic mutations can reveal clonal origins in non-dysplastic epithelium.

**What are the new findings?**

- Metaplastic columnar epithelium without goblet cells (‘cardia-type’) can undergo clonal expansion and harbour premalignant *TP53* mutations.
- Intestinal metaplasia is not required for clonal progression.
- Metaplastic columnar epithelium without goblet cells (‘cardia-type’) and columnar epithelium with goblet cells (‘intestinal metaplasia’) can derive from a shared clonal progenitor.

**How might it impact on clinical practice in the foreseeable future?**

- Our data formally establish the premalignant potential of columnar metaplasia without goblet cells in Barrett's oesophagus. These results provide fundamental insight into the histogenetic pathways of progression to oesophageal adenocarcinoma in Barrett's oesophagus. Our data do not mandate inclusion of patients with columnar metaplasia of the distal oesophagus without intestinal metaplasia on oesophageal biopsy into surveillance programmes in countries where this is currently not the norm.

Barrett's oesophagus (BO) is a common pre-neoplastic condition where the normal squamous epithelial lining of the distal end of the oesophagus is replaced by a mosaic of columnar epithelia with and without goblet cells in response to chronic acid–biliary reflux from the stomach.^1^ ^2^ Great controversy surrounds the issue of whether only the intestinalised columnar epithelium (‘intestinal metaplasia’) showing goblet cells can progress to oesophageal adenocarcinoma (OAC) or whether metaplastic columnar epithelium (often referred to as ‘cardia-type metaplasia’) without goblet cells can also progress to cancer in the context of BO. Currently, there is solid evidence of premalignant clonal expansion only for intestinal columnar epithelium.^3^ ^4^ Although circumstantial evidence has suggested premalignant potential of gastric columnar epithelium without goblet cell differentiation,^5–13^ there are no studies that have directly examined the progression of gastric columnar epithelium to OAC. Hence, the current American Gastroenterological Association and British Society of Gastroenterology guidelines state that there is insufficient evidence for tumour formation arising from this type of columnar epithelium.^1^ ^2^ Previous longitudinal studies have suggested that gastric columnar epithelium may develop intestinal features over time,^14^ but again direct genetic evidence in favour of a clonal relationship between these columnar epithelia has been lacking.

This gap in our knowledge surrounding the issue of whether premalignant progression in BO is exclusive to columnar metaplasia showing goblet cell differentiation impacts our understanding of the histopathological progression to cancer in the distal oesophagus. We reasoned that by reconstructing lineage relationships between columnar epithelia and OAC in an entire Barrett's segment through genetic lineage tracing we would be able to trace the clonal origins of OAC in this patient. By investigating the presence or absence of somatic point mutations, we can establish whether epithelial lineages or samples are ancestrally related; if two samples share a point mutation, then they must clonally derive from the same stem cell that once incurred this mutation. By comparing shared and divergent mutations we can build evolutionary trees, which recapitulate the evolution of a lesion.

Here we report a case where OAC evolved from non-dysplastic cardia-type columnar mucosa without goblet cell differentiation. Although this patient with BO demonstrated the characteristic mosaic of metaplastic columnar epithelia with and without goblet cells, the epithelium demonstrating intestinal metaplasia had clonally diverged from the premalignant expansion *prior to* the acquisition of oncogenic mutations. Together, these results demonstrate that tumour progression in the distal oesophagus is not exclusive to metaplastic columnar epithelium with goblet cells.

## Methods

### Spatial sampling

Patient is a 59-year-old female who underwent oesophagectomy following neoadjuvant radiochemotherapy. The resection specimen was transferred fresh to the pathology department immediately following surgery and opened according to standard operating procedure from distal to proximal. Longitudinal strips were cut along the length of the Barrett's segment from the squamocolumnar junction into the anatomic stomach. Tissue strips were mounted as ‘Swiss rolls’ in OCT compound for cryostat sectioning. Serial sections were prepared for H&E staining, cytochrome c oxidase (CCO) enzyme histochemistry, immunohistochemistry and laser capture microdissection (LCM). Material collection was carried out in accordance with locally and nationally applicable laws on tissue collection in The Netherlands (‘Code for proper Use of Human Tissue’, https://www.federa.org/).

### CCO enzyme histochemistry

Sequential histochemical staining for CCO, a component of complex IV of the respiratory chain enzyme, and succinate dehydrogenase histochemistry, a component of complex II of the respiratory chain (the presence of which was used to highlight the absence of CCO activity) was carried out on 10 μm cryostat sections according to published protocols.^15^ Briefly, sections were first incubated in CCO incubation medium (100 μM cytochrome c/4 mM diaminobenzidine tetrahydrochloride/20 μg/mL catalase in 0.2 M phosphate buffer, pH 7.0) for 30 min at 37°C. These were then washed in phosphate-buffered saline (PBS), pH 7.4, for 5 min and incubated in SDH incubation medium (130 mM sodium succinate/200 μM phenazine methosulfate/1 mM sodium azide/1.5 mM nitroblue tetrazolium in 0.2 M phosphate buffer, pH 7.0) for 30 min at 37°C. Sections were washed in PBS for 5 min, dehydrated in a graded ethanol series (70%, 95% and 100%) and left to air dry for 1 h before LCM.

### Deep mitochondrial DNA sequencing

CCO-deficient epithelium and stromal DNA were laser capture-microdissected (Zeiss, Munich, Germany) from cryostat sections. Total DNA extraction was carried out using QIAamp DNA extraction kit (Qiagen, Hilden, Germany) and quantified using Qubit dsDNA HS Assay kit (Life Technologies, Paisley, UK) according to the manufacturer’s instruction. Mitochondrial DNA (mtDNA) was amplified in two overlapping fragments using high-fidelity Takara LA Taq (Clontech, Saint-Germain-en-Laye, France); all reactions were performed in duplicate. DNA products were checked for the correct size and quantified for subsequent normalisation to 0.2 ng/μL using a 2200 TapeStation instrument (Agilent, Stockport, UK). Dual indexed libraries were generated from 1 ng of PCR products using Nextera XT library preparation technology (Illumina, San Diego, California, USA) and sequenced on the Illumina MiSeq platform according to the manufacturer’s recommendations. Variants in the mtDNA mutations were called from aligned sequence reads using deepSNV software, with only those reads with good concordance between duplicates taken forward for analysis. Somatic variants were identified by comparison with consensus-filtered calls from matched stroma.

### Immunohistochemistry

Immunohistochemistry on frozen sections was performed according to standard protocols. Details are available on request.

### Exome sequencing

We focused on single glands because we have recently shown that individual Barrett's glands are clonally derived units and thus define clonal boundaries along the Barrett's segment.^16^ Glands were individually microdissected using a laser capture microscope (Zeiss). Whole-genome amplification (WGA) was performed directly on LCM-captured glands using a single-step procedure, essentially as described.^17^ In short, single LCM glands were incubated for 10 min in Repli-g D2 buffer (Qiagen, Valencia, California, USA) and then in Repli-g Stop Solution. Glands were then mixed with Repli-g single-cell kit Master Mix and incubated at 30°C for 16 h in a thermocycler. Total DNA extraction was carried out using QIAamp DNA extraction kit (Qiagen, Hilden, Germany) and quantified using Qubit dsDNA BR Assay kit (Life Technologies, Paisley, UK), according to manufacturer’s instruction. Dual indexed libraries were generated using Nextera Rapid Capture (V.2) library preparation technology (Illumina) according to standard protocols and sequenced on an Illumina HiSeq, according to the manufacturer’s recommendations.

### Somatic mutation calling and phylogenetic reconstruction

Exome capture libraries were sequenced on an Ilumina HiSeq. Sequencing reads were aligned using BWA and further processed according to the GATK best practices pipeline. Mutations were called using MultiSNV and phylogenetic tree reconstruction was carried out using the maximum parsimony mode of the MEGA software.^18^ ^19^ Due to allelic amplification biases introduced by the Repli-g WGA step, copy-number profiling of the samples using the sequenza software was not deemed opportune and was not included in the analysis. Further details are provided in the online supplementary appendix supplementary figures 1 and 2.

### Sanger resequencing

Sanger resequencing was performed according to standard protocols using the BigDye terminator cycle sequencing method on an ABI Prism Genetic Analyzer (Applied Biosystems, Foster City, California, USA). mtDNA and *TP53* exon 8 primer sequences are available on request.

## Results

**Figure 1:**
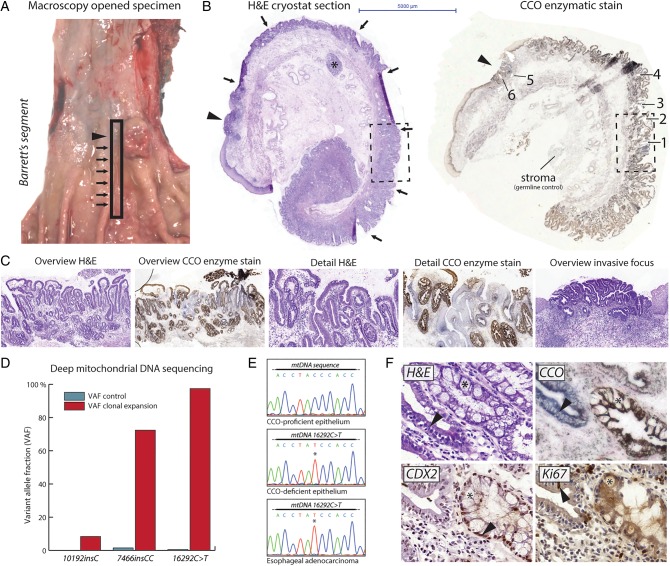
Spatial sampling of a Barrett's segment and associated oesophageal adenocarcinoma (OAC). (A) Overview of the opened resection specimen shows columnar metaplasia across the gastro-oesophageal junction (arrows) and a nodular OAC (arrowhead). The rectangular box indicates the longitudinal strip that was sampled. (B) H&E-stained cryostat section (left) of the longitudinal strip across the gastro-oesophageal junction reveals columnar metaplasia of the distal oesophagus (arrows) and an OAC at the squamocolumnar junction (arrowhead). Submucosal gland complex (asterisk) confirms the oesophageal origin. Cytochrome c oxidase (CCO) staining of this longitudinal strip (right) shows several discontinuous epithelial patches that are CCO-deficient. The OAC is also CCO-deficient. (B) and (C) images are taken at same magnification (see scalebar). The boxed area is shown in detail in (C). (C) Overview and high-power photomicrographs of one CCO-deficient epithelial patch and the associated OAC. Note the mosaic spread of the CCO-deficient clone within the background mucosa. There are no architectural or cytological features of dysplasia. OAC shows atypical, cribriform glands, which penetrate the pre-existent muscularis mucosae. (D) Deep next-generation mitochondrial DNA (mtDNA) sequencing reveals unique mtDNA mutations within the CCO-deficient epithelial patch shown in (C). Barchart shows the variant allele fractions (VAFs) of the mutations in control (stroma) sample and in material from the clonal expansion, mtDNA mutations are indicated. The *16290C>T* mutation was selected for further analysis. (E) Sanger resequencing shows that spatially distinct CCO-deficient epithelial patches and the OAC carry the same *16290C>T* mtDNA mutation (see also table 1). CCO-proficient epithelium does not carry this genetic lineage marker. (F) Consecutive sections of neighbouring glands showing cardia-type metaplasia and intestinal metaplasia. Top left: H&E staining shows absence of goblet cell differentiation in non-dysplastic cardia-type epithelium (marked by arrowhead), whereas the neighbouring intestinal metaplasia shows abundant goblet cells (marked by asterisk). Top right: clonal loss of CCO activity in cardia-type epithelium (marked by arrowhead). Bottom left: CDX2 staining confirms absence of intestinalisation in CCO-deficient cardia-type metaplasia. Strong nuclear labelling is seen in neighbouring intestinal metaplasia (marked by arrowhead). Bottom right: low proliferative activity as shown by Ki67 proliferation marker stain, consistent with morphological absence of dysplasia. Arrowhead points to positive nuclear labelling.

We examined a 59-year-old female who underwent oesophagectomy following neoadjuvant radiochemotherapy. The resection specimen revealed a nodular lesion on a background of columnar metaplasia of the tubular oesophagus (figure 1A). In order to investigate histogenetic clonal origins of OAC, we developed a spatial sampling technique that enabled detailed integration of histopathological and genetic analyses. By cutting thin, longitudinal mucosal strips running from the squamocolumnar junction into the anatomic stomach, we effectively captured the clonal and morphological evolution of columnar metaplasia and associated neoplasia in an entire Barrett's segment. H&E staining of cryostat sections of this snap-frozen mucosal strip revealed approximately 4 cm of columnar metaplasia of the distal oesophagus with a mosaic of metaplastic (‘cardia-type’) columnar epithelium without goblet cells and columnar epithelium with goblet cells (‘intestinal metaplasia’) (figure 1B,C). At the squamocolumnar junction, we observed a moderately differentiated OAC. Note that because the complete anatomical context is retained during tissue processing, we can confidently rule out that these abnormalities represent metaplasia and/or cancer of the cardia. We did not identify dysplastic precursor stages in this specimen.

**Table 1:** Overview lineage tracing results Sanger confirmation

Gland*	Histology	CCO staining	mtDNA resequencing	*TP53* status
1	Metaplastic columnar epithelium without goblet cells	Deficient	Mutant (*16290C>T*)	Heterozygous *TP53* mutation (*p.R273C*)
2	Metaplastic columnar epithelium with goblet cells	Proficient	Wild-type	Wild-type
3	Metaplastic columnar epithelium without goblet cells	Deficient	Mutant (*16290C>T*)	Heterozygous *TP53* mutation (*p.R273C*)
4	Metaplastic columnar epithelium with goblet cells	Proficient	Wild-type	Wild-type
5	Oesophageal adenocarcinoma	Deficient	Mutant (*16290C>T*)	Bi-allelic *TP53* mutation (*p.R273C*)
6	Oesophageal adenocarcinoma	Deficient	Mutant (*16290C>T*)	Bi-allelic *TP53* mutation (*p.R273C*)
NA	Stroma control	NA	Wild-type	Wild-type

*Refers to glands marked in figure 1B. CCO, cytochrome c oxidase; mtDNA, mitochondrial DNA.

To investigate clonal ancestry within the Barrett's segment, we performed CCO and succinate dehydrogenase enzyme histochemistry (‘CCO staining’) on frozen tissue sections of these mucosal strips. CCO staining reveals enzyme activity of the mitochondrial respiratory chain enzyme complex. The loss of epithelial substrate conversion indicates an underlying somatic mutation in the mtDNA. These somatic mtDNA mutations are effectively neutral (ie, do not alter proliferation or survival) and so delineate clonal expansions.^20^ Multiple CCO-deficient patches were observed indicating the presence of one or more clonal expansions within this mucosal strip (see figure 1B,C and online supplementary figure S3). H&E staining of serial sections showed that all these clonal expansions occurred in histopathologically non-dysplastic columnar mucosa (figure 1C). Importantly, the OAC at the squamocolumnar junction also showed loss of substrate conversion.

We performed deep next-generation mtDNA sequencing after LCM on material from one CCO-deficient clonal expansion to find genetic clonal markers. Sequencing reads were compared with reads from the underlying stroma to filter somatic mtDNA polymorphisms. This revealed a somatic mtDNA mutation (*16290C>T*) within the CCO-deficient clonal expansion at near (97%) homoplasmic levels (figure 1D). We then looked for the presence of the *16290C>T* mutation in the other CCO-deficient patches in this patient using LCM followed by Sanger sequencing. All CCO-deficient patches contained this same mutation, including the OAC, indicating that they shared a common clonal origin (figure 1E and table 1). Interestingly, this CCO-deficient clonal expansion was not contiguous along the Barrett's segment (see online supplementary figure S3), suggesting a complex pattern of clonal spread. Histopathological analysis of consecutive H&E and CCO-stained sections revealed that goblet cell differentiation was absent from all CCO-deficient epithelial patches, while CCO-proficient epithelial patches clearly showed goblet cell differentiation (figure 1C,F). Immunohistochemistry for CDX2, a marker of intestinal differentiation, on serial frozen sections confirmed this observation (figure 1F). Ki67 proliferation marker labelling showed absence of marked staining, consistent with the non-dysplastic features (figure 1F). These mtDNA lineage tracing studies document a clonal relationship between cardia-type columnar metaplasia and the associated OAC.

**Figure 2:**
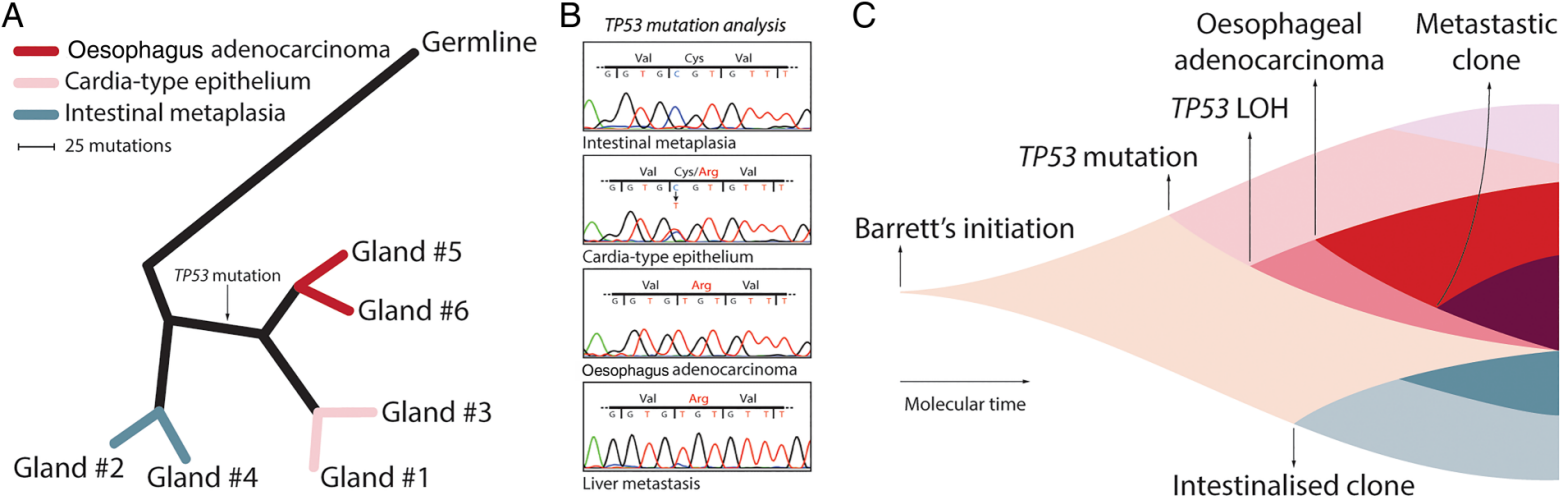
Clonal evolution of non-dysplastic metaplastic columnar epithelium without goblet cells to oesophageal adenocarcinoma. (A) Comparative exome sequencing was performed on glands along the Barrett's segment and of the oesophageal adenocarcinoma (marked in figure 1B). Phylogenetic tree shows the lineage relationships of glands sampled from the oesophageal adenocarcinoma (red), metaplastic cardia-type glands (pink) and intestinal metaplasia (grey). The adenocarcinoma branches from the cardia-type epithelium clade with which it shares a pathogenic *TP53* mutation, indicating premalignant progression. Leaf lengths not drawn to scale. (B) Sanger resequencing confirms the presence of a pathogenic *TP53* mutation (*c.817C>T; p.R273C*) in the metaplastic columnar epithelium without goblet cells, which is absent from neighbouring metaplastic columnar epithelium with goblet cells. The second *TP53* allele has undergone loss of heterozygosity in the oesophageal adenocarcinoma. This pattern is also detected in material from the liver metastasis, which presented 1 year after oesophagectomy. (C) Model for the clonal evolution of this oesophageal adenocarcinoma from metaplastic columnar epithelium without goblet cells in Barrett's oesophagus. Molecular time proceeds from left to right. Colour scheme is as shown in panel (A). Two metaplastic clones, which derive from a common progenitor, are detected within this Barrett's segment. Expansion of clones is associated with sub-clonal evolution. One subclone within the metaplastic columnar epithelium without goblet cells acquired a *TP53* mutation and eventually gave rise to metastatic oesophageal adenocarcinoma.

To further investigate the genetic phylogeny of non-dysplastic Barrett's mucosa and the associated OAC, we carried out multi-region whole-exome sequencing (WES) on single microdissected glands along this Barrett's segment. We built libraries from two spatially separated CCO-deficient glands (marked as gland no. 1 and gland no. 3 in figure 1B) and two random CCO-proficient glands showing goblet cell differentiation (glands no. 2 and no. 4 in figure 1B). In this way, we could directly compare mutational changes between clonally related and clonally distinct lineages. We also analysed two glands from the OAC at the squamocolumnar junction (glands no. 5 and no. 6). Sequencing reads were again compared with reads obtained from stroma to filter germline polymorphisms. From the mutational data in this set of six glands, we inferred a phylogenetic tree through maximum parsimony reconstruction (figure 2A). The phylogenetic tree obtained through single-gland WES analysis independently recapitulates the clonal ordering obtained from mtDNA mutation analysis and indicates a shared clonal origin of the cardia-type columnar metaplasia and the OAC. The glands showing goblet cell differentiation are also contained within one clonal expansion, but this clonal expansion clonally diverged *before* the non-dysplastic cardia-type columnar metaplasia underwent clonal expansion. Importantly, the clonal relationships derived from our WES analysis thus independently confirm the lineage relationships obtained through mtDNA mutation analysis.

When we examined the genes with non-synonymous somatic mutations shared between the glands sampled from the non-dysplastic clonal expansion in cardia-type epithelium (glands no. 1 and no. 3) and the OAC, we found that the only previously described OAC driver gene^21^ ^22^ mutated in this premalignant clonal expansion was *TP53* (table 2). Sanger resequencing confirmed that the non-dysplastic CCO-deficient cardia-type epithelium carried a monoallelic *TP53* point mutation (*c.817C>T; p.R273C*), consistent with pre-tumour genetic progression (figure 2B and table 1). This is one of the most common *TP53* transversions found in OAC, underscoring its pathogenic status.^23^ This mutation was not found in metaplastic glands showing goblet cell differentiation, confirming our WES results (figure 2B and table 1). This result formally demonstrates that premalignant clonal expansion in BO is not limited to columnar epithelium with goblet cells. The *TP53* mutation had undergone loss of heterozygosity in the associated OAC, again confirming our WES results. We also retrieved this oncogenic mutation in archival material obtained from a liver metastasis, which presented 1 year after oesophagectomy (figure 2B). To our knowledge this is the first time a metastatic clone of an epithelial cancer has been traced to its origin in non-dysplastic epithelium.

**Table 2:** Non-synonymous variants in COSMIC genes shared between the metaplastic columnar epithelium without goblet cells and the OAC

Gene	Chromosome	Position	Reference	Lineage
*ERCC3*	2	128030539	T	C
*ECT2L*	6	139186237	G	A
*AKAP9*	7	91691758	C	A
*MAML2*	11	95712231	T	C
*GOLGA5*	14	93301904	G	A
*TP53*	17	7577121	G	A
*NCOR1*	17	16049843	C	T

The *TP53* somatic variant is the only previously reported recurrent variant and was selected for resequencing. The majority of the variants used to derive the phylogeny of this patient's Barrett's segment are synonymous (see online supplementary figure S4).

OAC, oesophageal adenocarcinoma.

From these data we can reconstruct the clonal evolution towards OAC in this patient (figure 2C). Our data are consistent with a monoclonal origin of this Barrett's segment; from this initial clonal expansion, two lineages emerged; one of these later developed intestinal features, while the other retained a non-intestinal phenotype. The non-intestinal lineage sustained an oncogenic *TP53* mutation, which subsequently progressed to metastatic OAC. Our data conclusively demonstrate premalignant potential of metaplastic columnar epithelium without goblet cells in BO.

## Discussion

The population incidence and the magnitude of the risk of developing OAC in BO remain unsettled. A key factor in this uncertainty is the fact that there is no universally accepted (formal) definition of BO. On microscopic examination, BO often shows a mosaic distribution of metaplastic columnar epithelia with and without goblet cells. Of these metaplastic epithelia, only intestinal metaplasia is currently thought to progress to cancer in BO. In the USA, for this reason, only patients who show metaplastic columnar epithelium with goblet cells on histopathological examination of an oesophageal biopsy series fulfil the formal definition of BO. By contrast, in the UK, BO is primarily an endoscopic diagnosis and the presence of metaplastic columnar epithelium with goblet cells and Barrett's segment length together determine surveillance interval.^1^ ^2^ ^24^

We now present direct genetic evidence for the premalignant predisposition of metaplastic columnar epithelium without goblet cells in BO. Our spatial sampling strategy preserves all anatomic landmarks (such as submucosal oesophageal glands and double muscularis mucosae), allowing unambiguous definition of the columnar epithelium as metaplastic and the invasive lesion as a bona fide OAC. Detailed histopathological investigation coupled to independent genetic lineage tracing analyses across an entire Barrett's segment reconstructed the clonal ancestry of the OAC and revealed an ancestral premalignant clonal expansion in the non-dysplastic columnar epithelium without goblet cells.

Although previous epidemiological, histopathological and molecular studies had provided circumstantial evidence for the premalignant potential of metaplastic columnar epithelium without goblet cells BO,^5–13^ our study is the first to directly demonstrate that clonal expansion and premalignant progression in the context of BO is not exclusive to metaplastic columnar epithelium with goblet cells. We cannot rule out that premalignant progression of the metaplastic columnar epithelium without goblet cells was provoked or facilitated by the development of intestinal features in the neighbouring clone. However, the most parsimonious explanation for our data is that both lineages evolved independently. It is important to point out that our results do not challenge the clinical utility of goblet cells as a progression risk stratification marker. We do not advocate including all patients with columnar metaplasia of the distal oesophagus (ie, regardless of the presence of goblet cells) into clinical surveillance studies in countries where this is currently not the norm, since this would have substantial implications on resource usage.

We find that metaplastic columnar epithelium without goblet cells in BO can undergo clonal expansion and progression. *TP53* mutations have been previously described in non-dysplastic intestinalised Barrett's mucosa,^1^ ^3^ ^4^ but to our knowledge this is the first demonstration of a *TP53*-mutant clonal expansion in metaplastic columnar epithelium without goblet cells. We anticipated that neoadjuvant radiochemotherapy would surreptitiously induce genetic changes and indeed we observed widespread subclonal alterations at single-gland level in this patient (see online supplementary figure S4). Nonetheless, we could confidently retrieve the underlying phylogenetic signature from our WES data. This showed >90 somatic alterations which are shared between the metaplastic columnar epithelium without goblet cells and the OAC (see online supplementary figure S4). Note that while neoadjuvant radiochemotherapy will induce genetic changes, these are expected to be randomly distributed throughout the genome of each sample and are therefore unlikely to be shared between samples. By contrast, recurrent somatic variants (ie, shared between glands in this patient) are likely to have arisen *before* neoadjuvant radiochemotherapy. These shared variants allow us to reconstruct a genetic phylogeny.

Our approach allows us to map in detail the diversity of metaplastic columnar epithelia within one Barrett's segment. We deliberately studied a patient with the ‘classic’ mosaic patchwork of metaplastic epithelia to study the clonal relationships between these columnar metaplasias and the ancestral relationships of the OAC with these columnar epithelia. Our phylogenetic reconstruction indicates that the metaplastic columnar epithelium without goblet cells and the metaplastic columnar epithelium with goblet cells in this patient derive from a common precursor lineage. However, these lineages diverged *prior* to the development of the premalignant clonal expansion in the metaplastic columnar epithelium without goblet cells marked by the *TP53* non-synonymous variant. Previous studies have indicated that metaplastic columnar epithelium without goblet cells can show varying degrees of intestinalisation.^25^ ^26^ Note that none of the patches with goblet cell differentiation in this specimen showed loss of CCO staining (see online supplementary figure S3). Thus, while the premalignant clonal expansion in metaplastic columnar epithelium without goblet cells is *physically* bordered by metaplastic columnar epithelium with goblet cells, these epithelial clones represent phylogenetically divergent lineages and the intestinal metaplasia in this specimen did not clonally contribute to the OAC. This result supports our conclusion that although intestinal metaplasia is a risk marker in BO, it is not necessarily the precursor to cancer in BO.

The mosaic distribution of columnar epithelia that we analysed in this patient emulates the clinical problem of sampling error in BO. Our analyses reveal that the premalignant predisposition of columnar epithelium in BO is not exclusive to metaplastic columnar epithelium with goblet cells. This is an important step forward in our understanding of premalignant progression in the mosaic of metaplastic columnar phenotypes in the distal oesophagus of patients with BO. Although previous studies had shown that the presence of goblet cells on oesophageal biopsy is a marker of progression risk, what remained unclear is whether the development of goblet cells (within the expanding premalignant clone) was also required for progression. We now show that the development of goblet cells within the expanding clone is not required for tumour progression in BO. We also show that metaplastic columnar epithelium with goblet cells can clonally develop from metaplastic columnar epithelium without goblet cells. Collectively this indicates that in patients with BO, the entire Barrett's mosaic is at risk of transformation. This is supported by the fact that previous studies have shown that Barrett's segment length is an indicator of progression risk.^27^

In short, we demonstrate the premalignant nature of metaplastic columnar epithelium without goblet cells in the context of BO. A limitation of our study is the fact that we describe a single case. Future lineage tracing studies should evaluate what the relative risk of progression is in metaplastic columnar epithelium without goblet cells versus metaplastic columnar epithelium with goblet cells in patients with BO. Our results bring us a step closer to understanding the histopathological pathways of progression to OAC.

## Supplementary Material

Web supplement
