# Supplementary material for: Evolution of oesophageal adenocarcinoma from metaplastic columnar epithelium without goblet cells in Barrett's oesophagus
Source: Gut. 2015 Dec 23;65(6):907–13. doi: 10.1136/gutjnl-2015-310748 (PMC4893117; doi:10.1136/gutjnl-2015-310748)
Supplement: Web supplement [file gutjnl-2015-310748-s1.pdf]

Supplementary appendix

Supplementary Figure 1

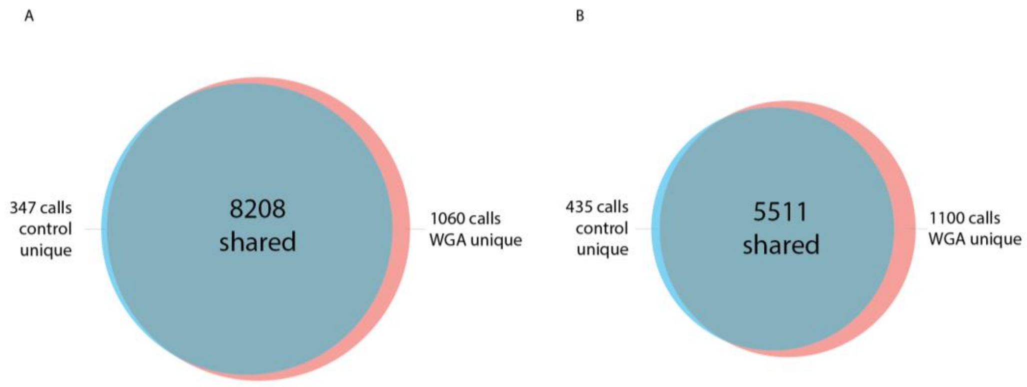

Supplementary Figure 1 shows that WGA faithfully reproduces the mutational complement of the target population. We validated WGA for point mutation detection by comparing WES libraries prepared from aliquots of bulk cell line DNA as a reference versus whole genome amplified cell line DNA (A) and WES libraries prepared from aliquots of DNA from needle-dissected frozen Barrett tissue as a reference versus whole genome amplified DNA from the same tissue section (B). Venn diagrams show that the concordance between WGA (pink) and unamplified libraries (blue) in both controls is > 85% (green overlap).

Supplementary Figure 2

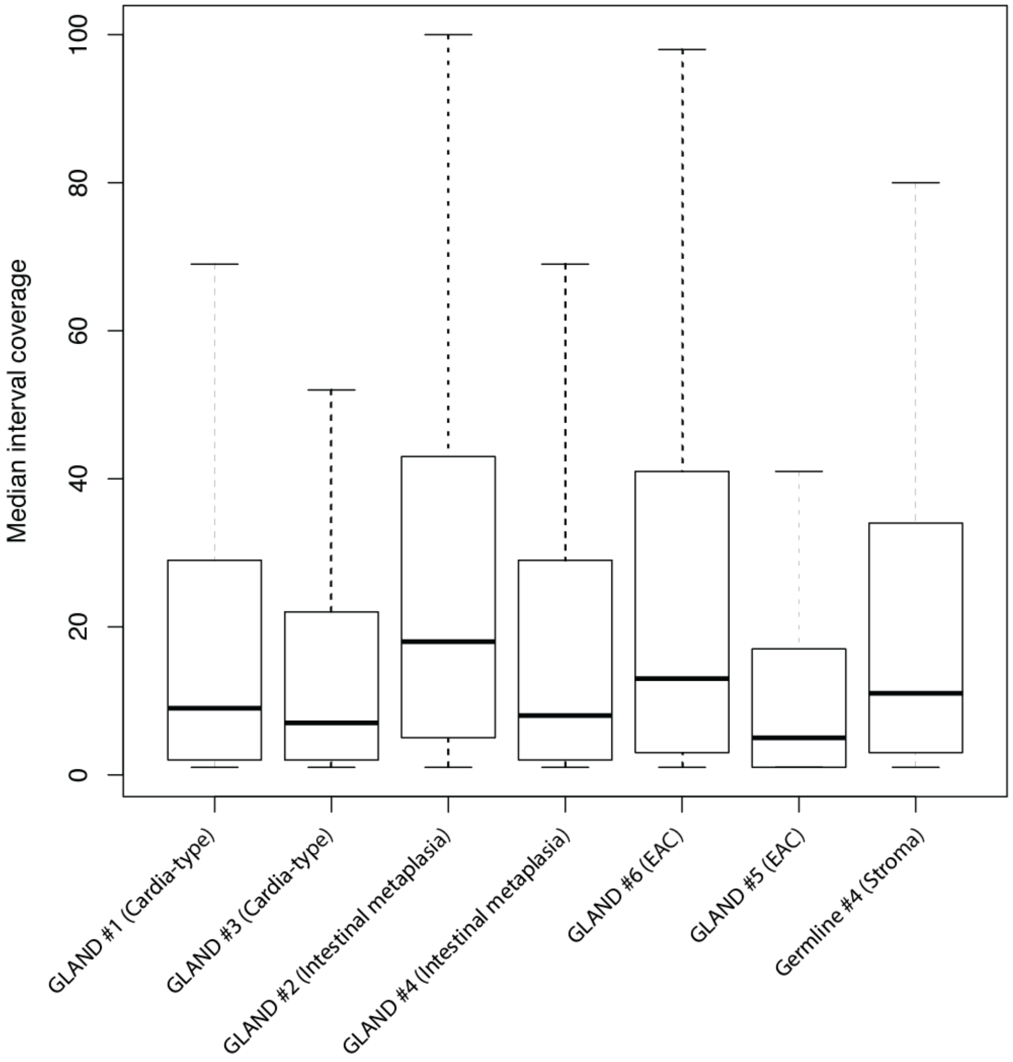

Supplementary Figure 2 shows the coverage statistics of the glands sequenced after WGA. As a consequence of the WGA procedure there will be inevitable unequal amplification of genomic regions. This will result in a general skew to a lower coverage with a long tail to regions that are sequenced at higher coverage. This artefact has been described by others sequencing small amounts of input material using this method as well (Lohr, J. G. et al, *Nature Biotechnology*, 2014, *32*; 479–484). However, microdissection of single glands results in clonal or quasi-clonal populations, which allows reliable detection of variants at shallow sequencing depth. Moreover, the goal of this experiment was not to perform an exhaustive search for possible driver variants, but to interrogate commonalities between samples to derive a phylogeny. The generally lower sensitivity therefore is not an issue, since the trees are built through a 'census-based' approach on somatic variants that are shared between samples. The final phylogenetic tree is independently supported by the mtDNA lineage tracing analysis and our resequencing experiments.

Supplementary Figure 3

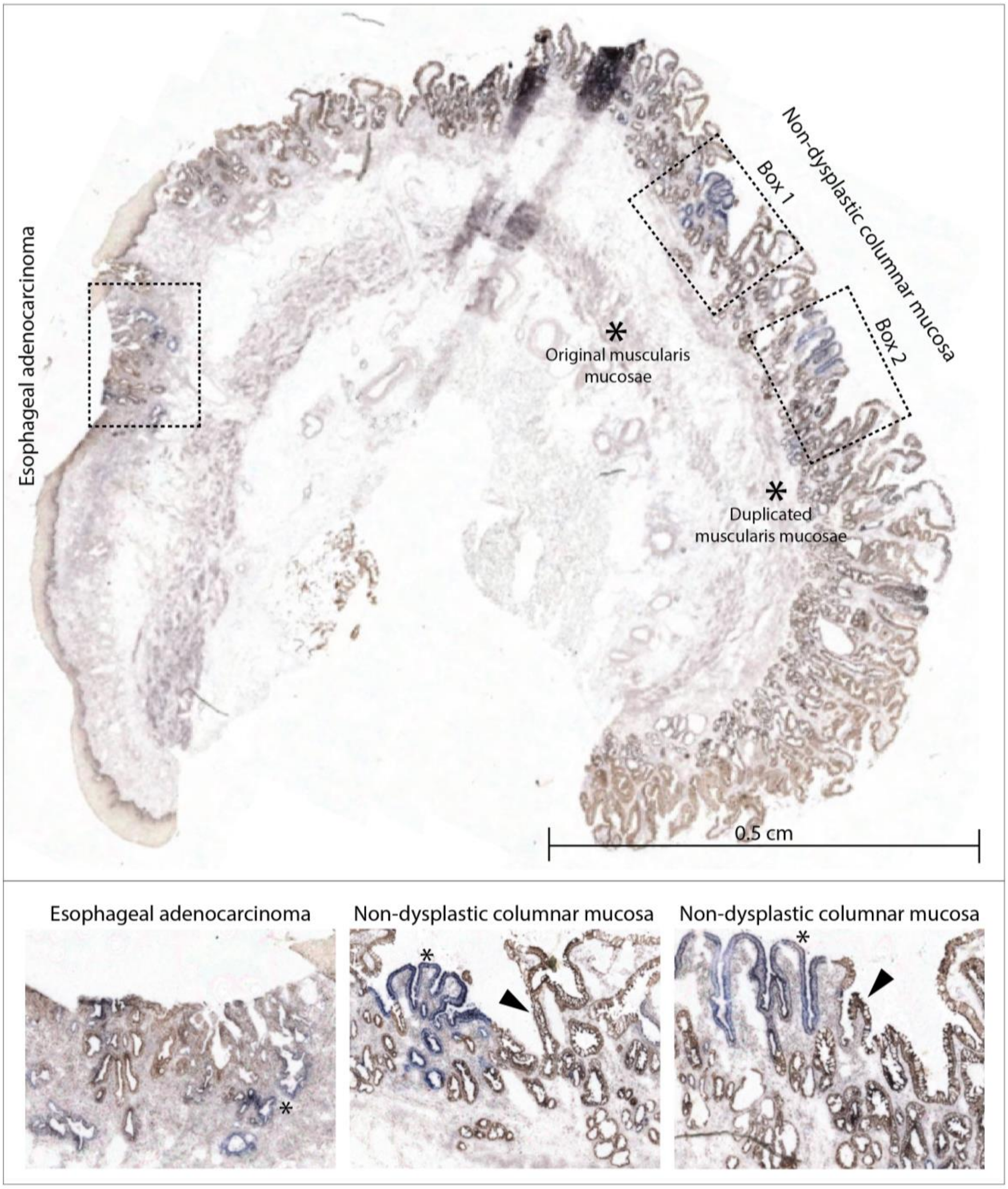

Supplementary Figure 3 shows a low-power overview of the complete CCO-stained Barrett’s segment. The CCO-deficient epithelial patches are marked (boxes) and shown at high-power in the bottom panels. Asterisks indicate the double muscularis mucosae re-emphasizing that this is metaplastic columnar mucosa in the anatomic oesophagus. Note the discontinuous pattern of clonal expansion. High-power photomicrographs are shown below. Asterisks mark the CCO-deficient clonal expansion; arrowheads mark background CCO-proficient intestinal metaplasia.

Supplementary Figure 4

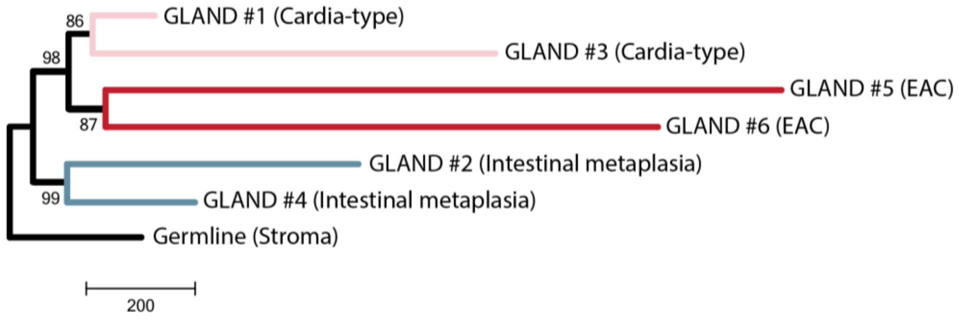

Supplementary Figure 4 shows the phylogenetic tree derived from maximum parsimony reconstruction. Neo-adjuvant pre-treatment and WGA together introduce single nucleotide variants, but the structure of the phylogenetic tree is not affected by the introduction of these variants. Indeed, this tree recapitulates the lineage relationships independently obtained from mtDNA analysis. Phenotypes are labelled on the individual terminal branches. The number of mutations (synonymous and non-synonymous) separating the branches is shown on the tree. The majority of mutations are synonymous. Of the genes with non-silent somatic mutations shared between the glands sampled from the non-dysplastic clonal expansion in cardia-type epithelium and the OAC (see Table 2) we selected the pathogenic *TP53* mutation for further analysis.
